# Very High Resolution Projections over Italy under different CMIP5 IPCC scenarios

**DOI:** 10.1038/s41597-023-02144-9

**Published:** 2023-04-26

**Authors:** Mario Raffa, Marianna Adinolfi, Alfredo Reder, Gian Franco Marras, Marco Mancini, Gabriella Scipione, Monia Santini, Paola Mercogliano

**Affiliations:** 1 Fondazione Centro Euro-Mediterraneo sui Cambiamenti Climatici, Regional Model and Geo-Hydrological Impacts (REMHI) Division, Via Thomas Alva Edison, 81100 Caserta, Italy; 2 HPC Department, CINECA, Via Magnanelli 6/3, 40033 Casalecchio di Reno, Italy; 3 Fondazione Centro Euro-Mediterraneo sui Cambiamenti Climatici, Advanced Scientific Computing (ASC) Division, Via Augusto Imperatore 16, 73100 Lecce, Italy; 4 Fondazione Centro Euro-Mediterraneo sui Cambiamenti Climatici, Impacts on Agriculture, Forests and Ecosystem Services (IAFES) Division, Viale Trieste 127, 01100 Viterbo, Italy

**Keywords:** Climate and Earth system modelling, Projection and prediction

## Abstract

This paper introduces VHR-PRO_IT (Very High-Resolution PROjections for ITaly), an open access hourly climate projection with a resolution of ≃2.2 km (i.e., Convection Permitting Scale) up to 2050, covering the Italian peninsula and some neighbouring areas. VHR-PRO_IT is produced within the Highlander project (https://highlanderproject.eu/) by dynamically downscaling the Italy8km-CM climate projection (spatial resolution ≃8 km; output frequency = 6 h; driven CMIP5 GCM = CMCC-CM) with the Regional Climate Model COSMO-CLM under the IPCC RCP4.5 and RCP8.5 scenarios. It covers the 60-year period 1989–2050. VHR-PRO_IT is intended for research purposes in the field of climate studies. For example, it may be included in the ongoing activities to clarify the added value of running climate simulation at the convection-permitting scale.

## Background & Summary

The rapid increase in computing power makes it possible to run regional climate models with kilometre-scale grids (i.e., <4 km) that allow convection to be solved explicitly on the model grid without needing a convective parameterisation scheme. These models are acknowledged as convection-permitting regional climate models (CP-RCMs). CP-RCMs are gaining broad attention from the climate community^1,2^, proving to be promising tools for representing hourly precipitation features (i.e. diurnal cycle, spatial structure, intensity distribution and extremes) and their sensitivity to climate change. In addition, improving spatial resolution allows the detection of surface heterogeneities (e.g. mountains, coastal regions and urban areas) and the simulation of land-atmosphere feedbacks necessary to preserve/amplify other extremes such as droughts or summer heat waves. These improvements can also knock on other variables (e.g. energy fluxes, such as latent and sensible heat, and soil moisture) relevant to different applications.

Some European initiatives, such as the Coordinated Regional Climate downscaling Experiment Flagship Pilot Study (CORDEX-FPS) on convection^3^ and the European Climate Prediction System (EUCP) project, and an increasing number of scientific works^4–12^ demonstrated the added value of CP-RCMs in reanalyses-driven simulations^4–9^ and climate projections^10–12^.

As part of the CORDEX-FPS convection initiative, Ban *et al*.^4^ and Pichelli *et al*.^12^ presented the first 10-year multi-model CP-RCMs ensembles at a horizontal grid spacing of ∼3 km for the greater Alpine region. Ban *et al*.^4^ showed that kilometre-scale models driven by ERA-Interim reanalysis^13^ reproduce daily and hourly precipitation more realistically than coarse-resolution RCMs, especially for extremes and frequency in the summer season. Pichelli *et al*.^12^ highlighted how CP-RCMs driven by CMIP5 Global Circulation Model (GCM) projections^14^ refine and enhance the patterns of change projected by coarse-resolution RCMs, even altering the sign of changes in intensity and extremes and returning positive changes in the frequency of extremes.

Recently, as part of the Highlander project (https://highlanderproject.eu/), Raffa *et al*.^15^ released VHR-REA_IT (Very High-Resolution REAnalysis for ITaly), a gridded dataset for the recent past thirty years (1989–2020) over Italy. Such a dataset has been obtained by dynamically downscaling ERA5 reanalysis^16^ from its native resolution (≃31 km) to a resolution of ≃2.2 km using the regional climate model COSMO-CLM^17^. VHR-REA_IT has been created to bring the potential of ERA5 to the convection-permitting scale. Its performance has been thoroughly evaluated against gridded observations to quantify its spatial and temporal added value for temperature and precipitation. Reder *et al*.^9^ adopted the same workflow, model, and configuration to produce ERA5@2 km, a high-resolution dataset for 20 European cities which provides data to estimate expected hourly precipitation at fixed return periods, adopted as input for pluvial flooding risk analysis^18^. ERA5@2 km has been evaluated against a set of observational datasets (comparable in spatial and temporal resolution), providing a more precise understanding of its added value in the localisation and magnitude of precipitation events at the urban scale, confirming the gain of CP-RCMs for the representation of extremes.

This work presents VHR-PRO_IT (Very High-Resolution PROjections for ITaly), an open access convective-scale climate projection up to 2050 covering the Italian peninsula and some neighbouring areas. VHR-PRO_IT is obtained within the Highlander project as a follow-up of VHR-REA_IT. It is produced by dynamically downscaling the Italy8km-CM climate projection^19,20^ (spatial resolution ≃8 km; output frequency = 6 h; driven CMIP5 GCM = CMCC-CM^21^) to the same spatial (≃2.2 km) and temporal (hourly) resolution of VHR-REA_IT. Its global forcing is the historical experiment driven by the observed natural and anthropogenic atmospheric composition for 1989–2005 and the RCP4.5 and RCP8.5 greenhouse gas concentration trajectories^22^ for 2006–2050.

Although it accounts for two greenhouse gas concentration trajectories, VHR-PRO_IT represents a single model projection of future climate change bypassing the effects of internal variability^23,24^ whose relevance is important when looked at geographically, especially for precipitation. It is mainly intended for research purposes in the field of climate studies. Its use in downstream applications (e.g. to support decision-making and adaptation) is not recommended due to the unavailability of a multi-model long-time CP-RCMs ensemble over Italy, allowing an adequate evaluation of uncertainties and robustness of its findings. Furthermore, applying bias-correction techniques^25–27^ to post-process data before their use in climate change impact or adaptation studies is strongly recommended to correct systematic biases against observations. Finally, it is worth noting that RCP4.5 can be considered likely under current policies. At the same time, RCP8.5, often called business as usual, should be clearly labelled as the unlikely worst case^28^, especially for climate impact studies. However, it remains relevant for research, as it was produced within the CORDEX initiative.

In any case, VHR-PRO_IT may represent a valuable dataset to investigate the added value of running CP-RCMs. The assessment of this added value is a goal of climate studies in recent years, as evidenced by different flagship activities (e.g. CORDEX FPS convection and EUCP) on such a topic. VHR-PRO_IT is expected to support this goal by understanding whether there is an added value in adopting CP-RCM climate projections in the different Italian contexts and supporting the development of cutting-edge approaches to exploit CP-RCM outputs. These issues are precisely the main ambitions of the Highlander project, where this climate projection was founded.

## Methods

### Regional Climate Model COSMO-CLM

COSMO-CLM^17^ is a non-hydrostatic limited-area model designed for climate simulations from the meso-β (~20–200 km) to meso-γ (~2–20 km) scale. It exploits finite difference methods to solve the governing equations of fully compressible fluid dynamics on a structured grid using finite difference methods.

Horizontal advection is calculated with a fifth-order upwind scheme, while vertical advection is calculated using an implicit Crank-Nicholson scheme^29^. Time integration is performed with a third-order split-explicit Runge-Kutta discretisation^30^. Cloud microphysics is modelled with a single-moment scheme^31^ using five hydrometeors (cloud water, rain, ice crystals, snow and graupel). The radiation scheme is based on a two-flow approach described by Ritter and Geleyn^32^. The turbulent fluxes within the planetary boundary layer are parameterised using a scheme based on turbulent kinetic energy (TKE)^33,34^. The Tiedtke mass-flux scheme^35^ is the default COSMO-CLM convective parameterisation. It is a mass-flux closure approach used to reproduce changes in the vertical structure of the atmosphere due to deep, mid-level and shallow convection. In the convection resolution setup (i.e. the one used for VHR-PRO_IT), only the surface convection part of the scheme is active, while the scheme for deeper clouds remains deactivated.

Soil moisture is modelled using the soil model TERRA_ML^36^ with a formulation for water runoff depending on orography. In addition, COSMO-CLM allows turning on the module TERRA-URB^37^ to simulate urban areas properly. TERRA-URB is a bulk scheme relying on a tile approach to discern for each grid cell between urban canopy and natural land cover and compute adjusted soil and water fluxes considering urban environment features. Such a module has been activated to produce VHR-PRO_IT data exploiting the very high horizontal resolution able to more detail urban covers.

### Climate simulation setup

**Fig. 1:**
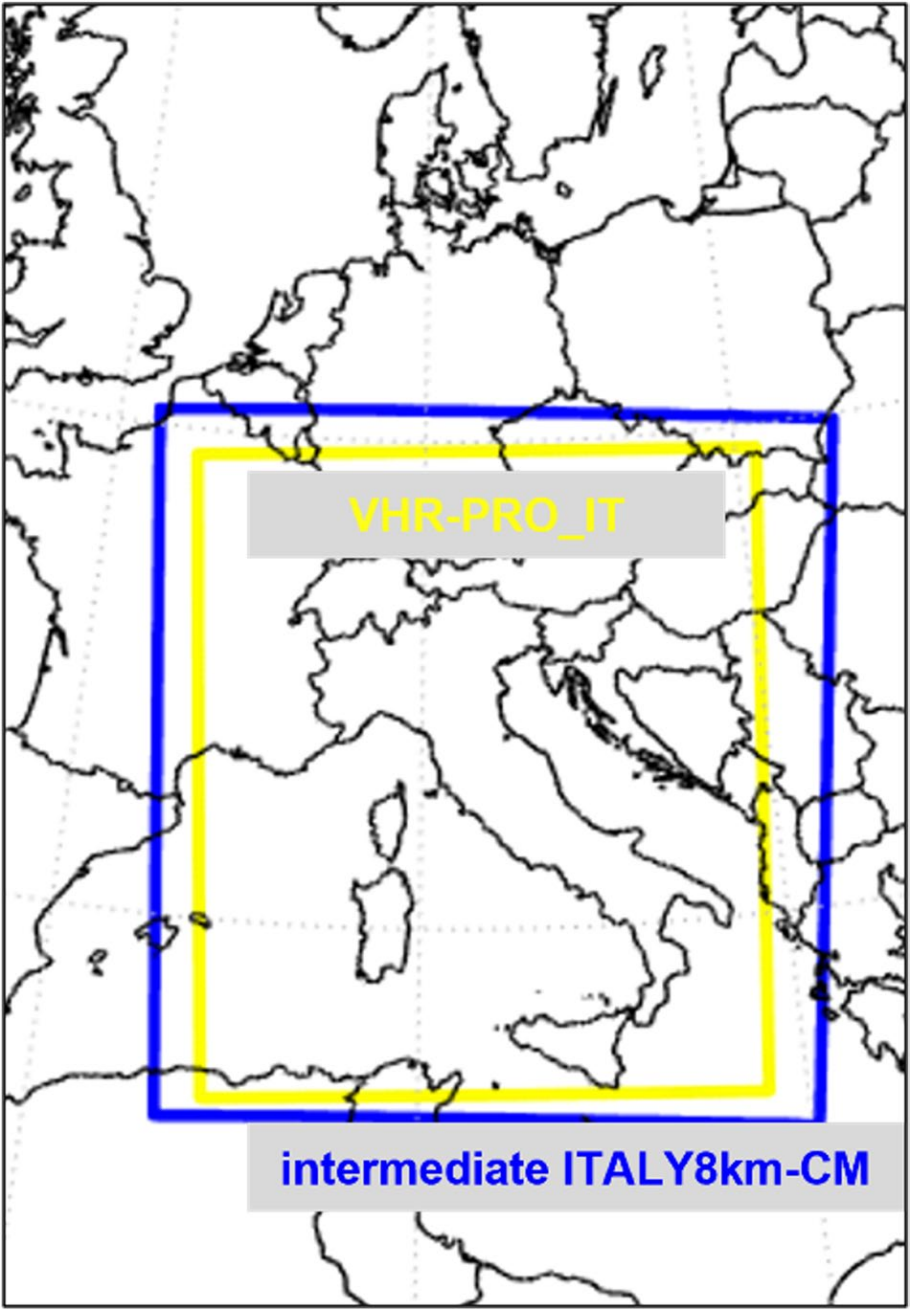
Computational domains of VHR-PRO_IT simulation and its driving model ITALY8km-CM.

The downscaling activity has been performed using the COSMO-CLM (v. CCLM5–0–9) turning on the module TERRA-URB (v. 2.3.1). The computational domain (Fig. 1) consists of 620 × 692 grid points with 50 vertical levels and 7 soil levels. On the other hand, the analysis domain is obtained by discarding the relaxation zone from the computational domain. It consists of 570 × 652 grid points and corresponds to the horizontal dimensions of the VHR-PRO_IT dataset.

The simulation covers the period 1989–2050 (i.e., 1989–2005 following the CMIP5 historical experiment, 2006–2050 under the IPCC RCP 4.5 and RCP8.5 scenarios), assuming the year 1988 as a spin-up. Its time step for integration based on a third-order Runge-Kutta scheme is 20 s. The model configuration is based on the COSMO-DE setup used by the Deutscher Wetterdienst (DWD) for numerical weather prediction applications. It has also been adopted by several institutes acting in the Climate Limited-area Modelling-Community as a reference for climate mode experiments in the frame of the CORDEX-FPS convection. COSMO-DE setup also is the same configuration employed for the downscaling of ERA5 to obtain VHR-REA_IT. Table 1 summarises the main features of the model configuration.

**Table 1:** Climate simulation configuration for VHR-PRO_IT.

Boundary forcing	ITALY8km-CM^19^ under IPCC RCP4.5 and RCP8.5 scenarios
Horizontal resolution	0.02° (≃2.2 km)
Time step	20 s
N° grid cells of the computational domain	620 × 692
N° vertical levels	50 (top model level elevation = 22 km)
Output frequency	1 h
N° grid cells of the analysis domain	570 × 652
Radiation scheme	Ritter and Geleyn^32^
Convection scheme	Shallow convection based on Tiedtke^35^
Cloud microphysics scheme	Single-moment scheme using five hydrometeors^31^
Land-surface scheme	TERRA-ML^36^ with TERRA-URB^37^ parameterisation
Land use	GLC2000^41^
Planetary boundary layer scheme	Mellor and Yamada^33^
Lateral Boundary Condition (LBC) update frequency	6 h
Soil initialisations	Soil temperature and soil moisture obtained by interpolation from ITALY8km-CM

### Climate simulation workflow

The simulation adopted the following workflow. Firstly, ITALY8km-CM has been interpolated to the rotated latitude-longitude grid of the COSMO-CLM through the INT2LM program. This tool provides the initial and boundary data necessary to run the COSMO-CLM. Generally, data from the global model GME (i.e., the icosahedral grid point model of DWD), the Integrated Forecasting System (IFS, i.e., the spectral model of ECMWF) and the regional COSMO-CLM itself, as in this case of ITALY8km-CM, can be processed directly, avoiding the pre-processing phase. Finally, a long-term climate simulation has been performed by setting an automatic restart procedure to prevent interruptions of simulation due to the maximum walltime of the SLURM (Simple Linux Utility for Resource Management) partition.

INT2LM and the COSMO-CLM are implemented for distributed memory parallel computers using the Message Passing Interface (MPI). A Makefile is provided with the source codes, where the compiler call, the options and the necessary libraries can be specified.

The Centro euro-Mediterraneo sui Cambiamenti Climatici (CMCC) Foundation performs the long-term climate simulation on the supercomputer cluster GALILEO100 (https://wiki.u-gov.it/confluence/display/SCAIUS/UG3.3%3A+GALILEO100+UserGuide) of the Consorzio Interuniversitario del Nord-Est per il Calcolo Automatico (CINECA). CINECA, as coordinator of the Highlander project, designed, set up and made all the necessary HPC and CLOUD infrastructure available. The HPC is equipped with 554 computing nodes with 48 cores. Each of them contains 2 × CPU × 86 Intel Xeon Platinum 8276–8276 L (24 cores at 2.4 GHz). All used computing nodes have 384 GB of memory.

The long-term simulation has been performed by exploiting 54 nodes, corresponding to 2484 cores, taking approximately 43 hours per simulation year. The best results are obtained by employing 46 of the 48 cores present in each node. About 12 million hours of HPC resources were used for the long-term simulation. A large amount of data (i.e., ≃16.5 TB of output data and greater than ≃53 TB of forcing data, including the 3-dimensional boundary data needed for the downscaling) was produced.

## Data Records

Table 2 provides a general overview of the main features of the VHR-PRO_IT dataset. The dataset contains hourly data on a rotated grid (≃2.2 km, irregular/rotated pole grid) with temporal coverage from 01/01/1989 00:00 to 31/12/2050 23:00 (i.e., 1989–2005 for the historical period; 2006–2050 for the future period). These data are delivered in NetCDF format (dimensions = time, longitude, latitude, single vertical level), generally on single levels (i.e., 2 or 10 meters from the surface depending on the selected variables), except for soil moisture available at seven soil levels (i.e., depth = 1, 3, 9, 27, 81, 243, 729 cm from the surface). The reference coordinate system is WGS84 (EPSG 4326). The file naming of the output variables is structured, following as much possible the “CORDEX approach”, as VariableName_DatasetName&Resolution_GCMModelName_CMIP5ExperimentName_RCMModelName&VersionID_Frequency_DDSid.nc

**Table 2:** Overview of the key characteristics of the VHR-PRO_IT dataset.

Dataset title	VHR-PRO_IT: Dynamical Downscaling with COSMO-CLM of historical (1989/2005) and future climate (2006/2050) data under scenario RCP8.5 at 2.2 km over Italy
Data type	Model-generated data (numerical) - Climate Projections
Dataset owner/provider	Fondazione Centro Euro-Mediterraneo sui Cambiamenti Climatici (CMCC)
Dimensions	time, longitude, latitude, single vertical level
Data input format	NetCDF (Climate and Forecast (CF) Metadata Convention; http://cfconventions.org/) both for input and output format. Exceptions under CF are however compliant with the UNIDATA Common Data Model (CDM) to codify the Coordinate Reference System (https://www.unidata.ucar.edu/software/netcdf-java/v4.6/CDM/index.html)
Data spatial structure	Rotated grid
Temporal coverage	01/01/1989 00:00 to 31/12/2050 23:00
Temporal resolution	1 h
Spatial extent (Horizontal coverage)	Latitude range: 36°N-49°N Longitude range: 3°E-20°E
Coordinate System	WGS84 EPSG 4326
Spatial resolution (Horizontal resolution)	≈2.2 km × 2.2 km (irregular/rotated pole grid)
Vertical coverage	Surface, 2 or 10 meters from the surface, or 1,3,9,27,81,243,729 cm depth depending on the variable
Vertical Resolution	All output variables are on single levels except soil moisture provided for 7 soil levels

Table 3  reports the list of the output variables (short and long name), measure units, a description of the meteorological fields, and the corresponding short name variable from the CMIP5 standard CORDEX.

**Table 3:** Overview and description of variables.

Long-Name	Short-Name	Units	Description	Correspondent CORDEX variable
2m temperature	T_2M	K	Temperature of air at 2 m above surface	tas
2m dew point temperature	TD_2M	K	Temperature to which the air, at 2 m above the surface, would have to be cooled for saturation to occur	tds
Total precipitation	TOT_PREC	kg m^−2^	Accumulated liquid and frozen water, comprising rain and snow, that falls to the surface	pr *
U-component of 10m wind	U_10M	m s^−1^	Eastward component of the 10m wind	uas
V-component of 10m wind	V_10M	m s^−1^	Northward component of the 10m wind	vas
2m maximum temperature	TMAX_2M	K	Maximum temperature of the air at 2 m above surface	tasmax
2m minimum temperature	TMIN_2M	K	Minimum temperature of the air at 2 m above surface	tasmin
Mean sea level pressure	PMSL	Pa	The pressure (force per unit area) of the atmosphere at the surface	psl
Specific humidity	QV_2M	kg kg^-1^	The mass fraction of water vapor in (moist) air	huss
Total cloud cover	CLCT	1	Proportion of a grid box covered by cloud; cloud fractions vary from 0 to 1	clt
Surface Evaporation	AEVAP_S	kg m^−2^	Accumulated amount of water that has evaporated from the surface	evspsbl *
Averaged surface net downward shortwave radiation	ASOB_S	W m^−2^	Amount of solar radiation (also known as shortwave radiation) that reaches a horizontal plane at the surface (both direct and diffuse) minus the amount reflected by the surface (which is governed by the albedo)	-
Averaged surface net downward longwave radiation	ATHB_S	W m^−2^	Thermal radiation (also known as longwave or terrestrial radiation) refers to radiation emitted by the atmosphere, clouds and the surface. This parameter is the difference between downward and upward thermal radiation at the surface	-
Surface snow amount	W_SNOW	m	Liquid water equivalent thickness of surface snow amount	snw *
Soil (multi-levels) water content	W_SO	m	Liquid water equivalent thickness of moisture content of soil layer	mrso *
Land-sea fraction	FR_LAND	1	Land Area Fraction	sftlf *
Surface height	HSURF	m	Surface Altitude	orog

* VHR-PRO_IT variable has to be converted to have the same measure unit of the corresponding CMIP5 standard CORDEX variable.

## Technical Validation

This Section investigates the robustness of the VHR-PRO_IT dataset in two different aspects:

1. model performance: statistical analysis for the historical period produced by comparing model data to reference observations to measure the model deviation from observations;
2. model consistency: statistical analysis for the future period developed by comparing model data to other climate projections to measure the model convergence towards a similar climate signal.

This analysis is performed at the daily scale for total precipitation and 2m-temperature, and at the hourly scale for total precipitation.

In particular, the analysis at the daily scale considers:

- as reference observations, the daily gridded dataset SCIA-ISPRA of the Italian Environmental Protection Agency (ISPRA), based on the interpolation of data from local weather stations, and available at ~5 km for temperature and ~ 10 km for precipitation;
- as additional climate projections, the Italy8km-CM climate projection^19^ and seventeen GCM + RCM (see Table 4) from the Euro-CORDEX initiative^38^ at ~12 km grid spacing under the IPCC RCP4.5 and RCP8.5 scenarios.

**Table 4:** List of climate projections selected from the EURO-CORDEX initiative.

Driving GCM (G)	RCM (R)	G	R	Acronym
CNRM-CERFACS-CM5	CLMcom-CLM-CCLM4-8-17	G1	R1	G1 + R1
	CNRM-ALADIN53		R2	G1 + R2
	RMIB-UGent-ALARO-0		R3	G1 + R3
	SMHI-RCA4		R4	G1 + R4
ICHEC-EC-EARTH	CLMcom-CLM-CCLM4-8-17	G2	R1	G2 + R1
	SMHI-RCA4		R4	G2 + R4
	KNMI-RACMO22E		R5	G2 + R5
	DMI-HIRHAM5		R6	G2 + R6
IPSL-CM5A-MR	IPSL-INERIS-WRF331F	G3	R7	G3 + R7
	SMHI-RCA4		R4	G3 + R4
MOHC-HadGEM2-ES	CLMcom-CLM-CCLM4-8-17	G4	R1	G4 + R1
	KNMI-RACMO22E		R5	G4 + R5
	SMHI-RCA4		R4	G4 + R4
MPI-M-MPI-ESM-LR	CLMcom-CLM-CCLM4-8-17	G5	R1	G5 + R1
	MPI-CSC-REMO2009		R8	G5 + R8
	SMHI-RCA4		R4	G5 + R4
NCC-NorESM1-M	DMI-HIRHAM5	G6	R6	G6 + R6

Conversely, the analysis at the hourly scale focuses on the summer season (June-July-August), as convective events and processes dominate this season for the region of interest. It considers:

- as reference observations, the hourly gridded dataset GRIPHO^39^ relying on the interpolation of data from local weather stations at ~ 10 km;
- as qualitative additional climate projections, the finding of the 10-year multi-model CP-RCMs climate projection ensembles at a horizontal grid spacing of ∼3 km provided by Pichelli *et al*.^12^ over the greater Alpine region under RCP8.5 scenario.

**Fig. 2:**
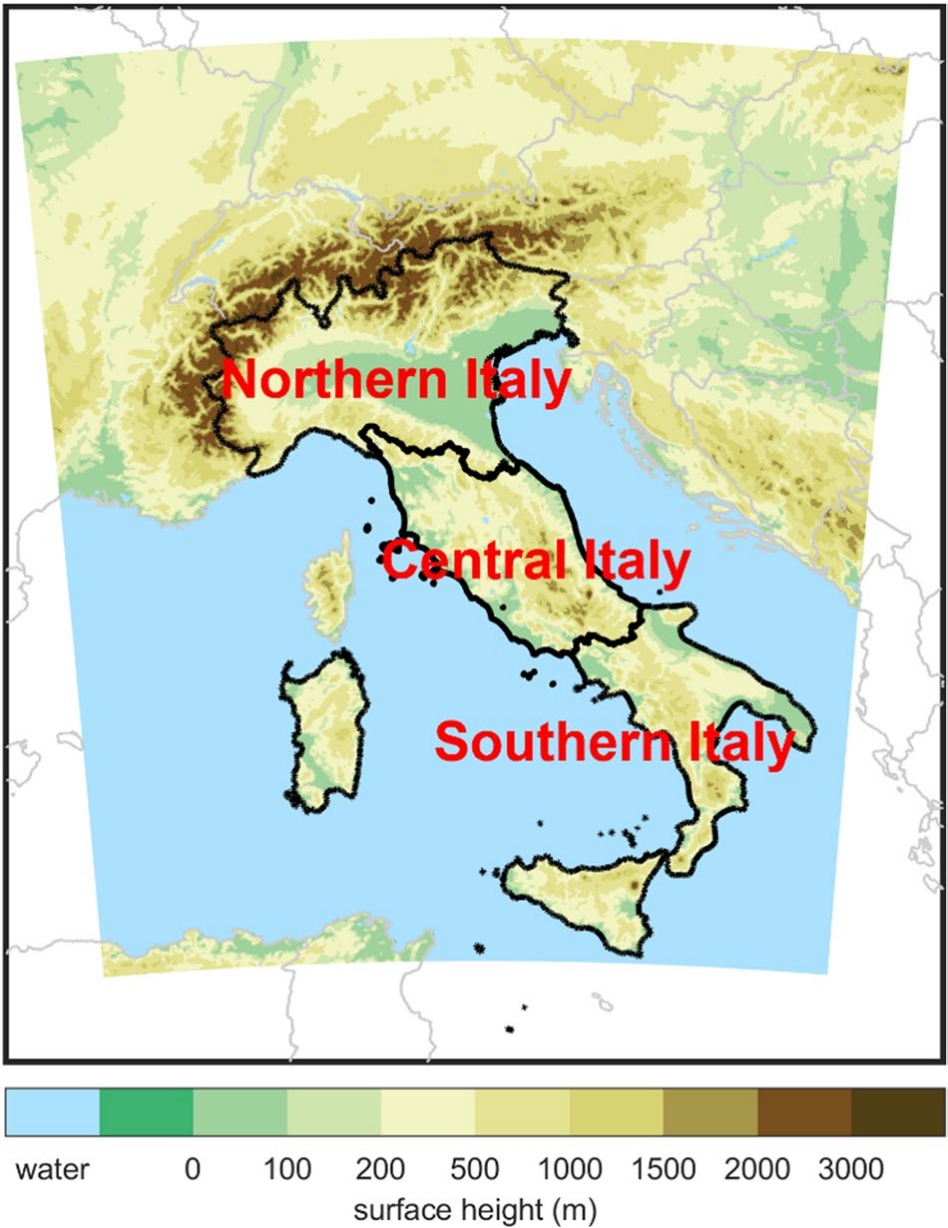
Surface height with highlighted Northern Italy, Central Italy and Southern Italy.

For both temporal scales, data are extracted and processed, separating Italy (see Fig. 2) into Northern Italy, Central Italy and Southern Italy, as done in Bucchignani *et al*.^19^.

### Analysis at the daily scale: model performance

**Fig. 3:**
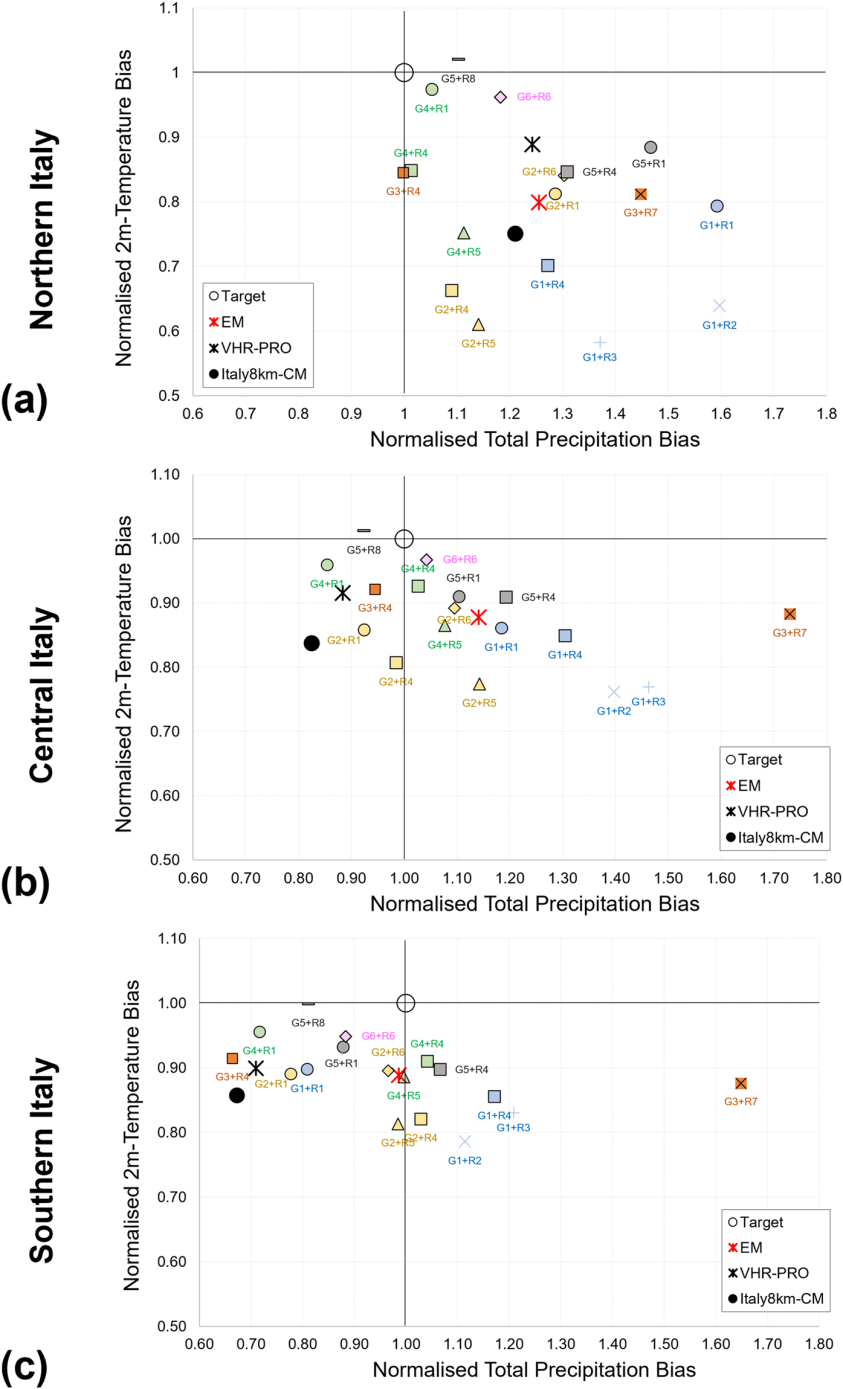
Model performance analysis over **(a)** northern, **(b)** central and **(c)** southern Italy. Each plot shows data as pairs of normalised precipitation bias against normalised temperature bias for VHR-PRO_IT, Italy8km-CM, and the Euro-CORDEX members with their Ensemble Mean (EM). Point (1,1) represents the observations as the target. Euro-CORDEX models are grouped according to GCM (same colours for each GCM) and RCM (same symbol for each RCM).

The model performance at the daily scale (see Fig. 3) is investigated over northern (Fig. 3a), central (Fig. 3b) and southern (Fig. 3c) Italy by assuming as indices the multi-annual average of 2m-temperature and total precipitation over 1989–2005. These indices have been first scaled to the observations and then represented in the plot normalised total precipitation – normalised temperature. The intention is to measure the distance between the pairs of values of each climate model from the target (1,1), representing the normalised observations. The lower the distance, the lower the bias of each model against observations and, thus, the higher its performance.

VHR-PRO_IT highlights a reduced simulation bias compared to the observations, demonstrating a satisfactory model performance over the historical period. Specifically, it outperforms Italy8km-CM reducing the bias for 2m-temperature and total precipitation (except over the Nothern Italy for the precipitation bias). Moreover, VHR-PRO_IT falls within the envelope of the Euro-CORDEX models, highlighting appropriate reliability. It returns a cold temperature bias w.r.t. the target, in line with all Euro-CORDEX members. On the other hand, it provides a dry precipitation bias in central and southern Italy and a wet precipitation bias in northern Italy. The sign of the precipitation bias agrees with the Euro-CORDEX members in the north and with more uncertainty in southern Italy. However, it is the opposite in central Italy, where most Euro-CORDEX models return a wet precipitation bias.

### Analysis at the daily scale: model consistency

**Fig. 4:**
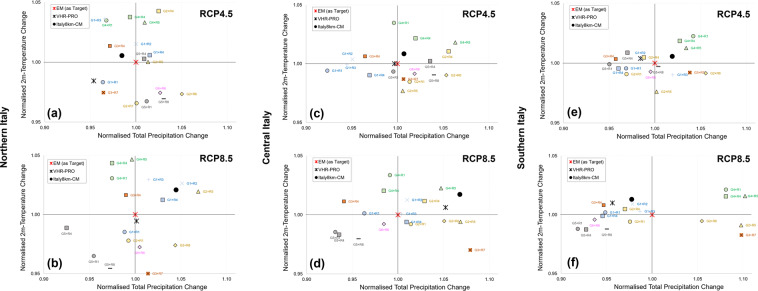
Model consistency analysis over (**a,b**) northern, (**c,d**) central and (**e,f**) southern Italy under (**a**–**e**) RCP4.5 and (**b**–**f**) RCP8.5 scenarios. Each plot shows data as pairs of normalised precipitation changes against normalised temperature changes for VHR-PRO_IT, Italy8km-CM, and Euro-CORDEX members. Point (1,1) represents the Euro-CORDEX Ensemble Mean (EM) as the target. Euro-CORDEX models are grouped according to GCM (same colours for each GMC) and RCM (same symbol for each RCM).

The model consistency at the daily scale (see Fig. 4) is investigated over northern (Fig. 4a,b), central (Fig. 4c,d) and southern (Fig. 4e,f) Italy under the RCP4.5 (Fig. 4a–e) and RCP8.5 (Fig. 4b–f) scenarios by assuming as indices the expected climate changes (2021–2050 vs 1989–2018) of the multi-annual average of 2m-temperature and total precipitation. Data over 1989–2018 are derived by combining historical data (1989–2005) with data for the period 2006–2018 from the RCP8.5 scenario. The indices have been first scaled to the ensemble mean (considered as the reference in terms of convergence toward a consistent climate signal) and then represented in the plot normalised total precipitation changes - normalised temperature changes. The aim is to retrieve the distance between the pairs of values of each model from the ensemble mean, i.e., the target point (1,1). The lower the distance, the higher the model consistency.

VHR-PRO_IT appears remarkably consistent with the Italy8km-CM projections and the EM of Euro-CORDEX models in terms of climate changes for 2m-temperature and total precipitation, falling within its envelope. The main differences are in northern Italy under RCP4.5 and central Italy under RCP8.5 and are mainly due to changes in total precipitation. Interestingly, the Euro-CORDEX members tend to cluster according to the GCM rather than the RCM, except for G3, featuring a divergent behaviour of R4 with respect to R7. In such clustering, the most consistent GCM turns out to be G1.

### Analysis at the hourly scale: model performance

**Fig. 5:**
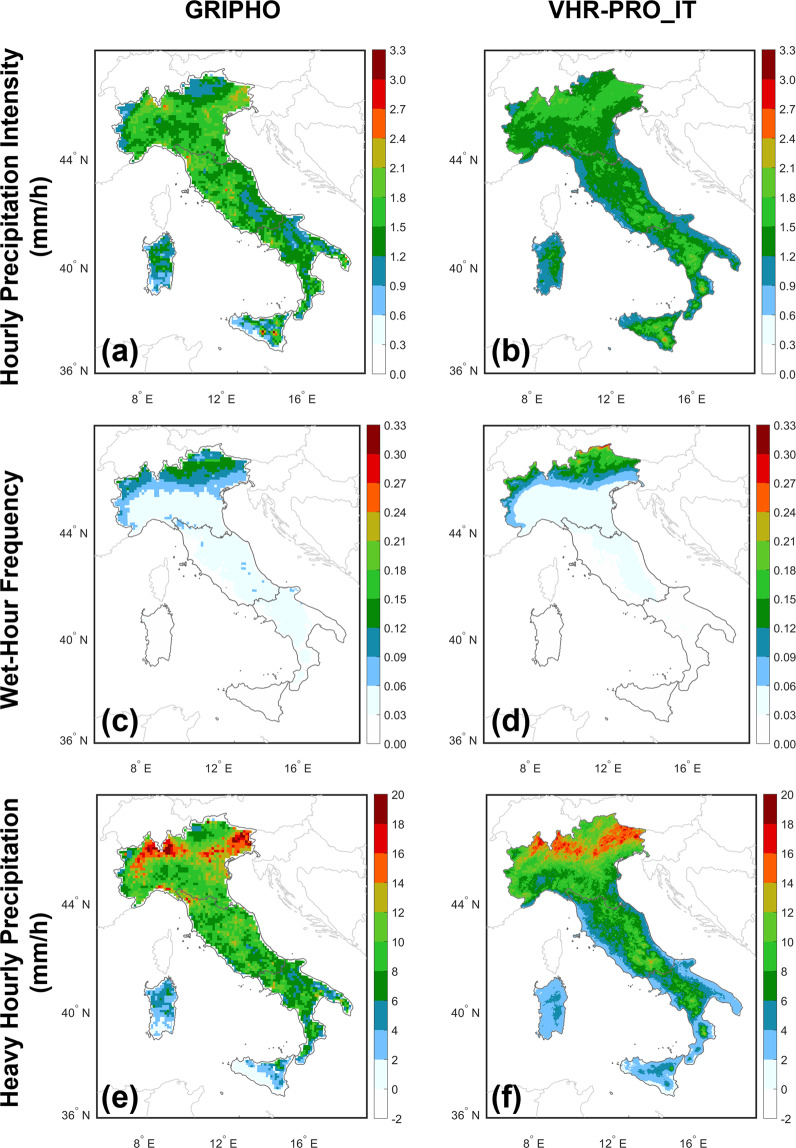
Summer hourly indices over Italy for (**a**–**e**) GRIPHO and (**b**–**f**) VHR-PRO_IT: (**a,b**) hourly precipitation intensity (mm/h), (**c,d**) wet-hour frequency, (**e**,**f**) heavy hourly precipitation (mm/h) events.

The model performance at the hourly scale (see Fig. 5) over the historical period is assessed as in Pichelli *et al*.^12^. The indices considered are mean summer wet-hour intensity (>0.1 mm/h, see Fig. 5a,b), wet-hour frequency (see Fig. 5c,d) and heavy hourly precipitation (i.e., the 99.9^th^ percentile of all events, see Fig. 5e,f). They are computed for VHR-PRO_IT over 1989–2005 (Fig. 5b–f) and GRIPHO over 2001–2010 (same period considered in Pichelli *et al*.^12^ for this dataset, see Fig. 5a–e) and qualitatively compared to the findings obtained by Pichelli *et al*.^12^ over the greater Alpine region (covering northern, central, and a small part of southern Italy) for the time slice 1996–2005. Although different periods are considered, this comparison aims to qualitatively assess the potential bias of VHR-PRO_IT against experiments with similar features assuming a shared reference (in this case, GRIPHO).

VHR-PRO_IT generally reproduces the summer hourly indices for the historical experiment well. Indeed, it agrees with observations considering the magnitude and spatial correlation, especially over complex orographic areas such as the Alpine region. Such an agreement aligns with the findings of the multi-model CP-RCMs climate projection ensembles reported in Pichelli *et al*.^12^.

Table 5 shows the biases of the analysed indices against GRIPHO in Northern, Central and Southern Italy. Biases are moderate for the areas examined (+1%/−13% for hourly precipitation intensity, −5%/−25% for heavy hourly precipitation). Compared to the same analyses conducted by Pichelli *et al*.^12^ over Italy (in an analysis domain incorporating northern, central and part of southern Italy), the frequency bias is practically the same. At the same time, a divergence occurs in the sign of the bias for the other two indices (i.e., intensity and heavy precipitation), which is mainly negative in VHR-PRO_IT and mainly positive in the multi-model CP-RCMs climate projection ensembles of Pichelli *et al*.^12^. In any case, in both experiences, the biases are significantly limited.

**Table 5:** Biases (model–observations) of VHR-PRO_IT (historical experiment) w.r.t GRIPHO (observations) for hourly precipitation intensity (INT-h), wet-hour frequency (FREQ-h) and heavy hourly precipitation (P99.9-h) averaged over northern, central and southern Italy.

	INT-h (mm/h)	FREQ-h (-)	P99.9-h (mm/h)
Northern Italy	−0.10	0.003	−0.69
Central Italy	−0.19	−0.010	−2.13
Southern Italy	0.01	−0.008	−1.30

### Analysis at the hourly scale: model consistency

**Fig. 6:**
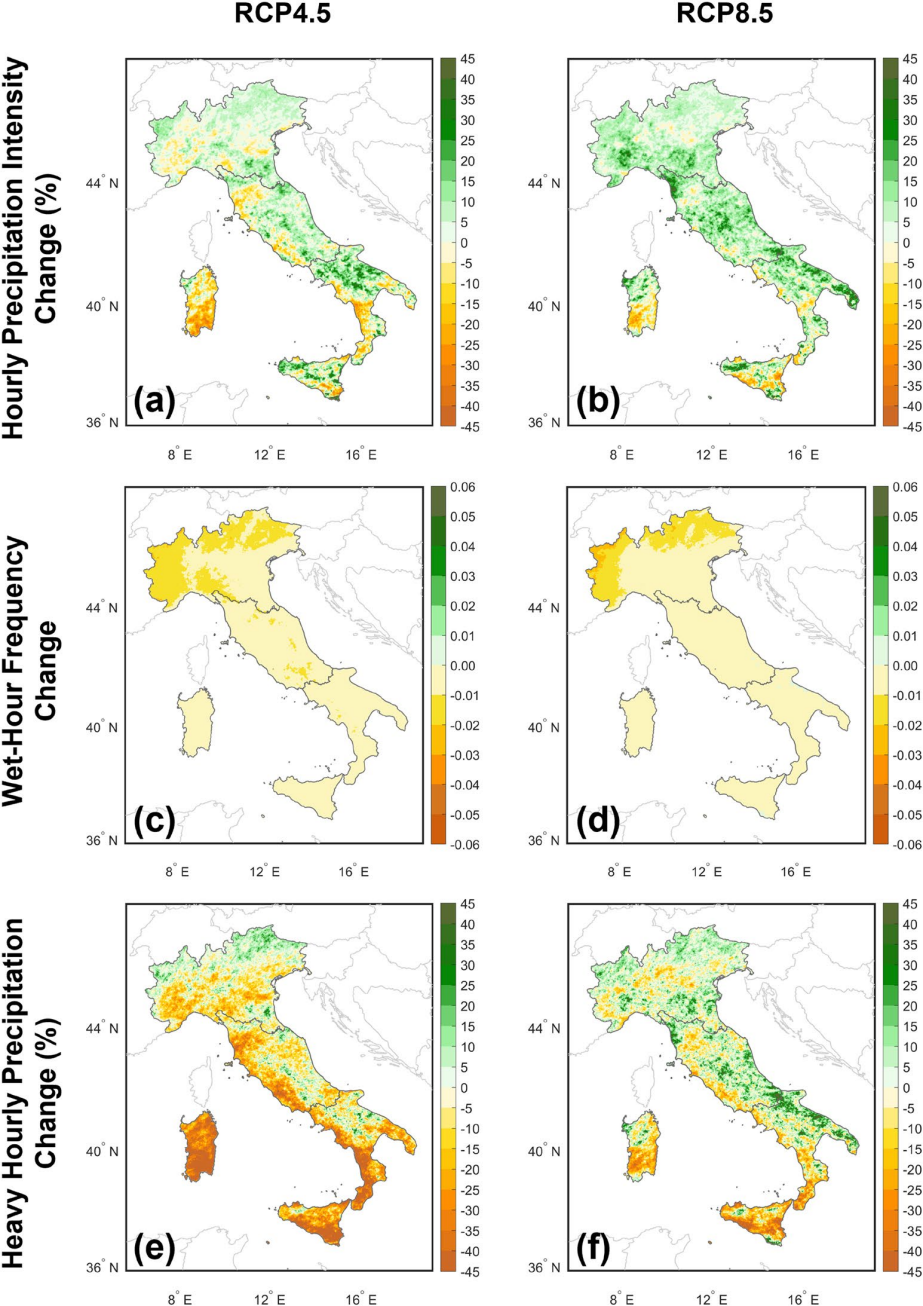
Mean change of the indices analysed over 2021–2050 for the summer hourly precipitation (reference = 1989–2018 obtained as historical for 1989–2005 + RCP8.5 for 2006–2018): (**a**,**b**) intensity change (percentage), (**c**,**d**) frequency change (absolute) and (**e-f**) heavy precipitation change (percentage). The results are obtained under (**a**–**e**) RCP4.5 and (**b**–**f**) RCP8.5 scenarios.

The model consistency at the hourly scale (see Fig. 6) is investigated considering the expected changes (2021–2050 vs 1989–2018) of the same indices adopted for the previous Section under the RCP4.5 (Fig. 6a–e) and RCP 8.5 (Fig. 6b–f) scenarios. Data over 1989–2018 are derived by combining historical data (1989–2005) with data for the period 2006–2018 from the RCP8.5 scenario. In addition, Table 6 shows the changes for the analysed indices averaged over Northern, Central and Southern Italy. Again, the changes reported in Pichelli for the multi-model CP-RCMs climate projection ensembles under the RCP8.5 scenario (end-of-century against historical periods) are taken as a comparison for an initial assessment of uncertainty.

**Table 6:** Changes (2021–2050 against 1989–2018) of VHR-PRO_IT under RCP4.5 and RCP8.5 scenarios for hourly precipitation intensity (INT-h), wet-hour frequency (FREQ-h) and heavy hourly precipitation (P99.9-h) averaged over northern, central and southern Italy.

	RCP4.5			RCP8.5		
	INT-h	FREQ-h	P99.9-h	INT-h	FREQ-h	P99.9-h
Northern Italy	3.5%	−0.010	−2.7%	7.3%	−0.009	3.5%
Central Italy	3.8%	−0.007	−10.0%	10.0%	−0.003	3.7%
Southern Italy	1.2%	−0.005	−25.0%	5.0%	−0.002	−6.0%

The values are percentage change [(future-reference)/(reference)] for INT-h and P99.9-h, and absolute change [future-reference] for FREQ-h.

The change in precipitation intensity is mainly positive in both scenarios, with the magnitude depending on the RCP considered (higher increase for RCP8.5 than RCP4.5). Conversely, frequency changes are negative across Italy, with minimum values over complex orography contexts (e.g. the Alpine region) and reduced for RCP8.5 compared to RCP4.5. This more intense but less frequent event signal is consistent with the Pichelli *et al*.^12^ multi-model CP-RCMs climate projection ensemble under the RCP8.5 scenario and each ensemble member.

Finally, changes in heavy precipitation are mainly related to the analysed scenario. For RCP8.5, an increase in extreme events intensity occurs over the Alps, Po Valley and the eastern Mediterranean, with modest decreases elsewhere, except for the insular regions (i.e., Sicilia and Sardinia) where reductions are remarkable. The RCP4.5 scenario preserves the same spatial distribution of the signal as for RCP8.5. Still, the values are rather reduced, as noticeable in the western Mediterranean (especially over insular regions and southern Italy) and Po Valley. The expected changes in heavy precipitation align with the spread of the Pichelli *et al*.^12^ multi-model CP-RCMs climate projection ensemble.

## Usage Notes

The dataset is available at CMCC at 10.25424/CMCC-J90A-5P12^40^. Data are stored at the CMCC Supercomputing Centre facilities and integrated into the CMCC Data Delivery System (DDS) (http://dds.cmcc.it), a unique, consistent, seamless access point for data produced by CMCC. The DDS user interface allows users to easily compile queries related to the dataset, by selecting a variable, a geographical area or location, and a period, and downloading data through the unified DDS API Python client interface. The file naming of the output variables is structured, following as possible the “CORDEX style”, as VariableName_DatasetName&Resolution_GCMModelName_CMIP5ExperimentName_RCMModelName&VersionID_Frequency_DDSid.nc (i.e., TD_2M_VHR-PRO_IT2km_CMCC-CM_rcp45_CCLM5-0-9_1hr_118902.nc). The download of the output variables via Python allows modifications in the file names of the data, according to the user requierements. The data are also available on the Highlander platform (https://highlanderproject.eu/data), which can be accessed similarly to the DDS service.

The data is in NetCDF format. Other standard interoperable formats (i.e., ESRI grid, GEOTIFF) can be provided on request for a sub-selection of the dataset, whose size will be defined according to the specific user needs and the processing time required. The easiest and fastest way to use NetCDF format is via command/script-based languages, such as CDO (Climate Data Operators) and NCO (NetCDF Operators), or via Python, Matlab and R. Some commercial GIS packages, such as ArcGIS (version 9.2 onwards), QGIS and IDRISI Taiga, allow the reading and processing of data. In addition, viewers, such as Panoply, ncBrowse, ncview and nCDF_Browser, allow simple data visualisation and map production.

Please cite this manuscript when using the VHR-PRO_IT dataset or part of it. In addition, please contact the corresponding author for any questions, suggestions or collaboration requests regarding the VHR-PRO_IT dataset.

## Code availability and data license

Data are protected by copyright. Distribution and communication of the data to the public are not allowed without the Fondazione CMCC’s written authorisation. Access to, consultation with, and reproduction of the data, in whole or in part, for personal use or institutional and research purposes is permitted, but not for commercial purposes and not with the intent to distribute, communicate, or make them available to the public. CINECA, through the Highlander platforms, and CMCC are the only institutions with permission to distribute, communicate, or make the data available to the public.

Adapting and transforming the data to create derivative works based on them, on the condition that an adequate mention of paternity is recognised through the citation of this paper to provide a link to the dataset Doi (10.25424/CMCC-J90A-5P12).

CMCC Foundation submits its data to adequate verification activities. However, CMCC Foundation does not assume responsibility for any inaccuracy or omission in them. CMCC Foundation is not responsible for the data and news published when processed by third parties and for the contents provided by any other site starting from such data. CMCC Foundation does not assume responsibility for any decision based on such data, which remains the sole responsibility of the user, nor for any loss or damage, direct or indirect, that may arise from their use.
